# Elucidating the mechanisms of TDP-43 aggregation in a cellular model of motor neuron disease

**DOI:** 10.1186/2193-1801-4-S1-L23

**Published:** 2015-06-12

**Authors:** David Hicks, Marcus Rattray

**Affiliations:** University of Bradford, UK

**Keywords:** TDP-43, MND, aggregation

Motor neuron disease (MND) is a neurodegenerative disease of mid-life, with average survival of 3-5 years after diagnosis. The only approved treatment, riluzole, is only moderately effective and so there is a great need for novel therapeutics. TAR DNA binding protein 43 (TDP-43) has held a particular centrality in MND research since its discovery as the principal protein component of the characteristic protein aggregates found in the disease. Subsequent work has investigated its role as an RNA-binding protein and regulator of approximately 3000 RNA transcripts. In the majority of disease cases, both familial and sporadic, normally nuclear TDP-43 is sequestered in the cytoplasm in protein aggregates. Using differentiated motor neuron-like cells (NSC-34) and primary motor neurons, we have treated cells with the disease-relevant ER stressor tunicamycin. Overnight treatment with a low concentration of tunicamycin resulted in the formation of aggregates immunoreactive for endogenous TDP-43, as visualised by immunocytochemistry and Western blotting of the RIPA-insoluble fraction. We have found that these aggregates do not co-stain for TIAR, a well-known marker of stress granules. However, using the oxidative stressor sodium arsenite, we are able to induce formation of stress granules, though they do not co-stain for TDP-43. The tunicamycin treatment also led to a moderate decrease in cell viability, as shown by MTT and Trypan Blue Exclusion assays. In this study, we have recapitulated a known pathological process (ER stress) of MND in vitro, which resulted in the formation of TDP-43 aggregates. The signalling pathways driving TDP-43 aggregation are unknown, hence work detailing these mechanisms is likely to present wide scope for therapeutic intervention.

